# Understanding the factors affecting attrition and intention to leave of health extension workers: a mixed methods study in Ethiopia

**DOI:** 10.1186/s12960-022-00716-1

**Published:** 2022-02-19

**Authors:** Merhawi Gebremedhin Tekle, Habtamu Milkias Wolde, Girmay Medhin, Alula M. Teklu, Yibeltal Kiflie Alemayehu, Esie Gebrewahd Gebre, Frehiwot Bekele, Nikita Arora

**Affiliations:** 1 School of Public Health, Haramaya University, Harar, Ethiopia; 2 Federal Ministry of Health, Addis Ababa, Ethiopia; 3 Addis Ababa University, Addis Ababa, Ethiopia; 4 MERQ Consultant PLC, Addis Ababa, Ethiopia; 5 Jimma University, Jimma, Ethiopia; 6 School of Geography and Environmental Studies, Haramaya University, Harar, Ethiopia; 7 Faculty of Public Health and Policy, London School of Hygiene and Tropical Medicine, University of London, London, United Kingdom

**Keywords:** Attrition, Intention to leave, HEWs, Ethiopia

## Abstract

**Background:**

The Health Extension Program (HEP) is Ethiopia’s flagship community health program, launched in 2003. Health Extension Workers (HEWs) are key vehicles for the delivery of the HEP. While it is believed that there is high attrition among HEWs, the magnitude of or reasons for attrition is unknown. Their intention to leave their jobs in the next 5 years has also never been investigated on a national scale. This study aimed to assess the magnitude of, and factors affecting HEWs’ attrition and intention to leave in Ethiopia.

**Methods:**

The study used mixed methods to address the research objectives**.** Using stratified random sampling and regions as strata, 85 districts from nine regions were randomly selected in Ethiopia. Within each study district, six kebeles (village clusters) were randomly selected, and all HEWs working in these kebeles were interviewed to capture their 5-year intention to leave. The study team developed a data-extraction tool for a rapid review of district-level documents covering the period June 30, 2004 through June 30, 2019 to gather their attrition figures. We used survival analysis to model attrition data and checked model goodness-of-fit using the Cox–Snell residual test. We additionally collected qualitative data from HEWs who had left their positions.

**Results:**

The attrition of HEWS over the lifespan of the HEP was 21.1% (95% CI 17.5–25.3%), and the median time to exit from HEWs workforce was 5.8 years. The incidence rate was 3.1% [95% CI 2.8–3.4]. The risk of attrition was lower amongst HEWs with level four certifications, with children, and among those working in urban settings. By contrast, HEWs who were not certified with a certificate of competency (COC), who were deployed after 2008, and those who were diploma/degree holders were more likely to exit the HEWs workforce. The magnitude of intention to leave was 39.5% (95% CI 32.5–47%) and the primary reasons to leave were low incentives, dearth of career development opportunities (50.8%), high workload (24.2%), and other psychosocial factors (25%).

**Conclusion:**

Although the magnitude of attrition is not worryingly high, we see high magnitude in HEWs’ intention to leave, indicating a dissatisfied workforce. Multiple factors have contributed to attrition and intention to leave, the prevalence of many of which can be reduced to fit the needs of this workforce and to retain them for the sustained delivery of primary healthcare in the country. Ensuring HEWs’ job satisfaction is important and linked with their career development and potentially higher rates of retention.

## Background

As a strategy to improve the delivery of health services and reduce the overall inequity in access to primary health care in Ethiopia, the Health Extension Program (HEP) was launched as a supply-side reform in Ethiopia in 2003 [1, 2]. The HEP was initially piloted in select rural areas in four of the country’s nine regional states, which are home to over 80% of the country’s population, and later expanded to the rest of the country by 2009 [3, 4]. As part of the program, a package of primary health services is provided by trained, salaried, Community Health Workers (CHWs) called Health Extension Workers (HEWs), who are normally based in health facilities called health posts (HPs) [5, 6]. They form the second largest (16%) labor force in the Ethiopian health care service [7], and 21% of the recurrent health budget is spent on their salaries [4].

In Ethiopia, there is no published evidence on the magnitude of HEWs’ attrition or intention to leave. The intention to leave of a workforce is important, and theorists such as Ajzen [8] have sought to explain factors that predict actual turnover, through the theory of planned behavior/reasoned action, concluding that behavioral intention is the primary antecedent to actual behavior [9]. This implies that the cognitive process of intention to leave is an important predictor of actual turnover, a concept that has much empirical and theoretical support within labor market turnover research. Attrition, defined in this paper as HEWs’ exiting the workforce for any voluntary or involuntary reason, has been recognized as a potential threat to the sustainable delivery of the HEP.

As workforce shortages ensue and difficulties in retention are highlighted, it follows that the reasons HEWs leave their jobs must be clearly identified if the issue is to be successfully addressed. Job satisfaction has been cited as a major contributory factor to workforce exits [10, 11] and is an important determinant of health worker motivation, retention, and performance. In the context of CHWs, factors such as supportive supervision, clearly defined roles with specific tasks, locally relevant incentive systems that combine monetary and non-monetary incentives, recognition, training opportunities, community and policy support, and strong leadership [5, 12] have all been identified as determinants of job satisfaction and important to the success of the community health programs. All of these factors, in addition to individuals’ intrinsic motivation, can play a role in how long a worker serves as a CHW [13].

Previous studies around attrition have not been conducted on a national scale in Ethiopia. A few studies have been conducted using qualitative methods to understand the predictors of attrition [14–17], and financial issues, unsatisfactory working conditions, and a lack of career development have been ranked as top factors contributing to attrition and HEWs’ intention to leave [14, 18]. The high attrition of HEWs can be a barrier to providing sustainable and quality healthcare to the population [19, 20]. Recent government reports have described the attrition of HEWs as a challenge to the success of the HEP [21].

The development of effective, evidence-based planning of human resources for the HEP and the retention of HEWs should start from a concrete understanding of the magnitude of the problem and its leading reasons. Therefore, the primary objective of this study was to assess the magnitude of, and factors associated with the attrition and intention to leave of HEWs currently working as part of the heath system.

## Methods

### Study setting

Ethiopia has nine regions and two city administrations. In rural areas, the lowest government budget center is the woreda and the lowest administrative structure is the kebele, which has an average population size of 5000. There are about 4000 health centers (HCs), 400 public hospitals, and nearly 17 000 health posts (HPs) in the country. Two HEWs are responsible for a population of around 5000. After a year-long training on HEP components and health delivery packages, HEWs in rural areas, who are normally secondary-school graduates, are recruited to be deployed within the same community, where their permanent residence is registered [22]. HEWs in urban areas, on the other hand, are often diploma holders who receive a month-long training on HEP services and are deployed in urban settlements [5, 23–27]. In the hierarchy of professionalism in human resource cadres, HEWs are recruited at level three, which means that they are less skilled than level four workers including, nurse-midwives. HEWs must earn a certificate of competency (COC), a level-based examination provided for lower-skilled (i.e., below degree-level) professionals to matriculate to the next level of education and progress up the career ladder.

### Data collection and sampling procedure

The data used to develop this paper were collected in two rounds. The first round was carried out from February to May 2019, covering nine agrarian and pastoralist regions. A random sample of 584 HEWs was interviewed about their intention to leave their job in the next 5 years and whether they were actively looking for a new job at the time of the interview. The second round of data collection was conducted in June 2019, across all regional states and the two city administrations (Addis Ababa and Dire Dawa).

Data were collected using three methods: a retrospective review of deployment records of those ever employed as HEWs in randomly selected districts across the country to study attrition in the cadre; a nationally representative cross-sectional survey of HEWs currently working in the public sector to capture their intent to leave; and qualitative interviews with leavers of HEW positions to understand the drivers of attrition.

Data extracted from deployment records, covering the period between June 30, 2004 through to June 30, 2019 were used to determine the magnitude of and time to attrition. Using a cluster sampling technique, we selected 85 cluster sites that had HEWs posted in them; of which 64 were from the above-mentioned nine regions, 20 were from Addis Ababa, and one was from Dire Dawa. We reviewed the deployment records of all HEWs who had ever been deployed in the cluster as long as their personnel database was complete with the required information.

We conducted a cross-sectional survey with active HEWs in 64 woredas selected from the nine regional states to analyze their intention to leave. Within each of the selected woredas, six kebeles (village clusters) were randomly selected, and all HEWs working in these kebeles were contacted for the survey. Through this process, a total of 344 HPs were selected, and two HEWs per HP (688 in total) were approached to be interviewed.

To understand the reasons HEWs resign from their posts and the main drivers of attrition, we conducted qualitative in-depth interviews (IDIs) with HEWs who had left their jobs (“leavers”). We purposively sampled and interviewed 16 leavers who had recently left their jobs as HEWs. We also undertook a focus group discussion (FGD) with health administrators working at the regional and district levels to capture the perspectives of managers about the drivers of HEW attrition.

Research assistants experienced in quantitative data collection were recruited to administer the cross-sectional survey to respondents, while experienced researchers in qualitative research were recruited to undertake the IDIs and FGDs. All research assistants were recent graduates from public health schools within local universities who were provided 10 days of intensive training on the research before data collection commenced. Training was also provided on the survey tools that were designed to extract data from personnel documents and qualitative data collection. Chart reviewing was assisted by the human-resources personnel of the respective cluster sites. Data collection was performed using Open Data Kit (ODK) software (for quantitative data) and voice recorders (for qualitative data).

### Survey instruments and outcome variables

Attrition was our primary outcome variable and was calculated by estimating the number of officially deployed HEWs who had left their jobs for various reasons, including voluntary resignation, official dismissal, disappearance, death, retirement, change in qualification, and transfer. Their survival time in the job was calculated using the “date of deployment” and “date of attrition” for those who had left, and the date of data collection for censored cases (defined as HEWs who were in their posts during data collection period). The status of the respondent was recorded as a binary outcome, coded “zero” for those who were still in the job and “one” if they had left their jobs. Other information such as annual leave, competency of certification (COC) status, whether ever reprimanded by district officials, any recognition provided, and availability of career progression opportunities were also extracted from the deployment records.

To measure the intention to leave of active HEWs, we used a survey questionnaire consisting of close-ended questions about their intention to leave their jobs in the next 5 years. This survey included questions on intention to leave, along with possible timing and qualitative reasons they might want to leave their posts. Intention to leavef was another outcome variable and it was measured in two ways: first, by asking HEWs how many more years they intended to work as HEWs, and second, whether they were currently seeking another job. Using responses from these two questions, HEWs were defined as intending to leave if they said they intended to leave within the next 5 years or if they said they were actively currently searching for a job.

### Sampling weight

Sampling weight for attrition data was estimated based on the following two probabilities. First, the sampled district that was included from a given region was divided by the total districts available in the respective region. Second, the number of HEWs available in the given region was divided by the total HEWs available in Ethiopia. The two probabilities were multiplied, and the inverse of the result was taken as the final sampling weight to be included in the survey design.

### Data analysis

STATA version 14 (STATA Corporation, College Station, Texas 77845, USA) was used to analyze the quantitative data. Descriptive statistics were presented using means, medians, interquartile ranges (IQR), standard deviations, and proportions. Overall attrition among HEWs was calculated by dividing the number of HEWs who left their work by those who were deployed in posts over the 15-year study period. Person-year of observation (PYO), which is described as the total estimated time that all participants contributed to the study or remained at risk of attrition, was used as a denominator to calculate an incidence rate of attrition. It was calculated by summing up the number of persons who were at risk of attrition in each study year (15 years in total) excluding those who exited their jobs. The incidence rate of attrition was thus calculated by dividing the number of failures/attrition by person-year observation that HEWs remained at risk of attrition.

The duration of time and the number of HEWs who stayed in their jobs during the 15-year period of the study were modeled using survival analysis [28], which is commonly used to measure attrition or turnover within a workforce [14, 29, 30]. The survival time (ST) in a “year” and sampling weight were set in advance by indicating the “time-to-event” and “status” variables. Median and mean were calculated separately for failures and survivors, and time to exit from HEWs workforce was compared across strata of covariates. The survival function was calculated for the level of certification, adjusted for selective time marker covariates. Explanatory variables were pre-selected according to their hypothesized relevance after reviewing literature.

The Cox proportional hazards (PH) assumption was checked by a graphical and statistical approach. We use log–log Kaplan–Meier survival estimates of graphical presentation, where a parallel survival curve indicates that the assumption is not violated. Another graphical plot, called the Cox adjusted survival curve, was applied to see whether the Cox adjusted survival curve was close to the Kaplan–Meier survival curve. In other words, variables with a closer observation to the expected value indicate that the assumption is satisfied. A graphical proportional hazard assumption was carried out on 13 covariates, of which nine variables were fitted and entered into the “Cox test” to determine the equality of their survival curves.

A Cox PH model was used to estimate crude and adjusted hazard ratios at 95% confidence intervals (CIs) and *P* values < 0.05. Eight variables remained significant in the Cox test and were re-entered into the Cox PH model to determine the crude hazard ratio, of which seven variables fitted the model. To control for possible confounders, multivariate Cox PH model analysis was conducted on seven covariates, of which two variables, annual leave and family size, failed to fit the model. Finally, the five variables were re-tested in the Cox PH model, and five remained well fitted.

We also used the statistical approach to detect the assumption objectively. This assumption test was carried out after the final Cox model was performed; a global test ≥ 0.1 for all covariates indicated that the assumption is not violated. Cox statistical PH assumption was applied for the best-fitted model; a significant global test (*P* < 0.00) was observed on two variables (having children and deployment year), indicating that the application of an extended Cox model was needed. After using an extended Cox model on the two variables, the *P* value for time-varying-covariate (TVC) on “deployment year” was insignificant (*P* < 0.395), but it was significant for “having children” (*P* < 0.00). A Heaviside function was applied by taking survival time greater than or equal to 10 years as a reference point, and the *P* value for TVC was reported at *P* < 0.444.

Overall model goodness-of-fit was checked by a Cox–Snell residuals test, where residuals should have a standard censored exponential distribution with hazard ratio 1. If the model fits the data, the plot of the cumulative hazard and the Cox–Snell should approximate a straight line, with a slope equal to one [31].

### Qualitative data

For qualitative IDIs and FGDs, we developed separate topic guides to assess reasons for leaving their jobs among the former HEWs. Interviews were transcribed and translated into English, then exported to NVivo (v.12) software for analysis. Words, phrases, statements, and paragraphs were coded by one person and then rechecked by another member of the research team. Coded items were regrouped to create a theme, then themes were named by a common term that potentially included all codes. Themes were not finalized till both coders came to a consensus about the selected codes.

## Results

### Participant characteristics

A total of 3476 personnel deployment records of HEWs were reviewed, resulting in 2 809 023 person-years of observation. Of these, 96% were female, 56.4% were first appointed to their HPs when in the age range of 20–24 years, and 76.9% were single at the time of their deployment. More than half (57%) had one or more children, 54% were level three certified health workers, and 26% had ever upgraded to at least one level higher than their initial level of certification. Twenty-three percent of the HEWs had a history of administrative reprimands, 67% were COC certified, and, according to records, 42% had no documented evidence of having taken any annual leave during their time in service.

In the cross-sectional survey, a total of 584 HEWs responded to the study questionnaire. Of these HEWs, the majority (56%) were in the age range of 25–29 years, 76% were married, 71% had one or more children, 51% were level four certified, and 60% were COC certified. Table 1 summarizes respondent demographics.

**Table 1:** Socio-demographic and other characteristics of participants in the cross-sectional survey and record review

Variable	Category	HEW records reviewed *N* = 3476		HEW participants in cross-sectional survey *N* = 584	
		Unweighted frequency	Weighted percentage	Unweighted frequency	Weighted percentage
Sex	Male	85	4	0	0
	Female	3391	96	584	100
Age category	18–24	2835	79.8	189	21.7
	25–29	436	14.6	272	55.7
	30 and above	205	5.6	123	22.6
Marital status at deployment	Single	2697	76.9	184	24.3
	Married	734	22	400	75.7
	divorce/separated/widowed	29	1.1	0	0
Number of biological children	No children	1267	42.7	215	29.4
	One or more children	2030	57.3	369	70.6
Deployment year	2004–2008	1343	31.5	172	45.4
	2009–2013	983	30.4	158	27.9
	2014–2018	1150	38.1	254	26.7
Certification level	Level three or below	2086	54.4	345	48.9
	Level four	879	24.8	239	51.1
	Diploma/degree	495	20.8	0	0
COC status	COC certified	2347	67	332	60
	Not certified	1113	33	252	40
Reprimand status	Never reprimanded	2545	77.3	–	–
	Reprimanded once or more	915	22.7	–	–
Annual leave	No annual leave	1274	41.9	–	–
	1–3 annual leaves	1312	34.9	–	–
	> 3 annual leaves	873	23.2	–	–
Satisfaction level	Satisfied	–	–	304	45.3
	Not satisfied	–	–	280	54.7
Burnout	No burnout	–	–	144	15.6
	Little burnout	–	–	227	44.2
	Severe sign of burnout	–	–	213	40.2

Ethiopia, 2019

### Magnitude of attrition and intention to leave

Table 2 summarizes the magnitude of attrition and intention to leave among different strata of respondents. The average rate of attrition among all sampled HEWs in the 15 years of follow-up of the HEP was 21.1% (95% CI 17.5–25.3); it was lowest in the Harari region (6%) and highest in Addis Ababa (39%). The attrition rate was 36.7% for males, 22.5% in the 25–29-year age group, 21.9% among HEWs whose family size was 1–3, 32.4% for those who were COC uncertified, 33.5% for diploma holders, 21.7% among those who were married, 31.9% among pastoralist woredas, and 21.5% for those who were either never granted or had never requested annual leave since their hiring.

**Table 2:** Percentage of attrition and intention to leave by background. Ethiopia, 2019

Variable	Category	Attrition rate (*N* = 3476)	Intention to leave (*N* = 584)
		Weighted %	Weighted %
Region	Tigray	16.3	32.2
	Afar	36.1	42.1
	Amhara	25.3	64.2
	Oromia	15	40.3
	Ethiopian Somali	–	25.3
	Benshangul–Gumuz	–	21.6
	Southern Nations, Nationalities, and Peoples Region (SNNPR)	18.6	27.9
	Gambela	20	48.4
	Harari	6.1	58.8
	Addis Ababa	38.5	–
	Dire Dawa	8.4	–
	National	21.1	39.5
Woreda type	Agrarian	18.7	–
	Pastoralist	32	–
	Urban	21.2	–
Age category	18–24	21.4	35.2
	25–29	22.5	41.4
	30 and above	14.3	39.1
Deployment year	2004–2008	25.5	37.1
	2009–2013	27.9	46.4
	2014–2018	12.1	36.5
Certification level	Level three	21.2	36.9
	Level four	8.6	42
	Diploma/degree	33.5	0
Having biological children	No	24.1	43.2
	Yes	18.3	37.9
Family size	One to three	21.9	–
	Four or more	19.2	–
Certificate of competence status	COC certified	14.9	44.6
	Not certified	32.4	31.8
Recognition	No recognition	20.6	–
	One or more recognitions	20.9	–
Administrative reprimand	No reprimand	20	–
	At least one reprimand	22.9	–
Annual leave	No annual leave	21.5	–
	1–3 annual leaves	18	–
	≥ 4 annual leaves	23	–
Satisfaction level	Satisfied	–	31.4
	Not satisfied	–	46.3
Burnout	No burnout	–	27.3
	Little burnout	–	32.3
	Severe sign of burnout	–	52.2

Ethiopia, 2019

Intention to leave among active HEWs, on the other hand, stood higher, at 39.5% (95% CI 32.5–47%). It was lowest in Benshangul–Gumuz (21.6%) and highest in Amhara (64.2%). Intention to leave among HEWs between 25 and 29 years was 41.6% and 47.2% among non-married HEWs.

### Reasons for attrition and intention to leave

HEWs left their posts in different ways: 27.3% formally submitted a resignation letter; 40% simply disappeared without notice; 18.7% left their posts due to a change in qualification; 3% died; 4.7% were discharged from employment for various reasons, including absenteeism and poor work performance; and 6.6% were transferred out. Most HEWs who intended to leave in the next 5 years or were actively looking for other jobs mentioned low salary and limited career development as their reason (50.8%), while a smaller number (24.9%) mentioned psychosocial factors, such as family influence, health problems, and the need for safety, as reasons for their intention to leave. The workload was a reason for 24.2% of HEWs who intended to leave.

### Incidence rate and median time to attrition

The median time to exit from HEWs workforce was 5.8 years (IQR 2.9 to 8 years). The mean survival time for those who left was 5.7 (± 3.3 SD) years and 7.5 (+ 4.5 SD) years for those who were censored (i.e., who had not resigned), with a median time of 8 years.

The contribution of PYO was 2,809,023, and the overall incidence rate was 3.1% [95% CI 2.8–3.4%]. The highest incidence, 4.8% [4.8%; 95% CI 3.7–6.2%], was observed in the 7th year of employment. The incidence rate was lower among COC-certified HEWs, at 2.2% [95% CI 1.9–2.5%] compared to non-certified HEWs [4.4%, 95% CI 3.8–5.1%]. The incidence of attrition was higher among those who had no children, at 5% [95% CI 4.3–5.8%], than those with one or more children, at 2.1% [95% CI 1.9–2.4%]. Those who had been deployed recently (since 2013) had a higher incidence rate, at 4.1% [95% CI 3.3–5.3%] compared to those deployed earlier in the program (i.e., before 2008, at 2.3% [95% CI 1.9–2.6%]). The lowest rate of incidence was observed among level four HEWs, at 0.9% [95% CI 0.7–1.2%] and highest among diploma/degree holders, at 6.6% [95% CI 5.7–7.7%]. Those working in remote woredas had an incidence rate of 3.6% [95% CI 3.0–4.4%], and for those working in nearby woredas, it was 2.9% [95% CI 2.6–3.4%].

**Fig. 1:**
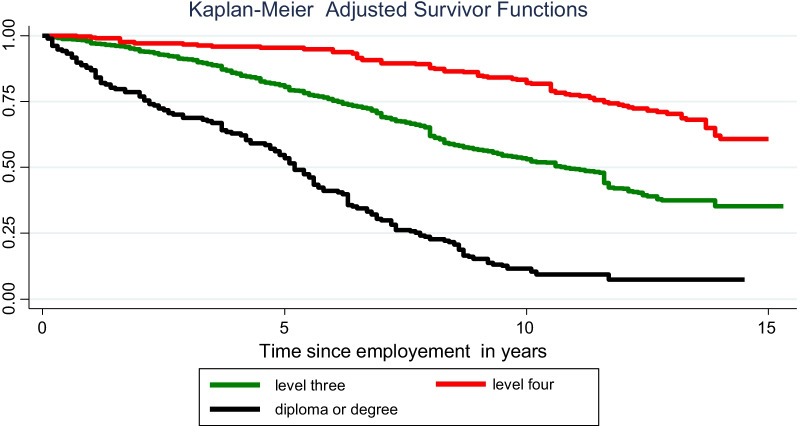
Survival function of HEWs by level of certification adjusted for other variables. Ethiopia, 2019

Figure 1 gives the Kaplan–Meier survival function, calculated to determine the survival of HEWs by the level of certification, which was adjusted for COC, number of children, year of deployment, and woreda type.

### Predictors of attrition

**Fig. 2:**
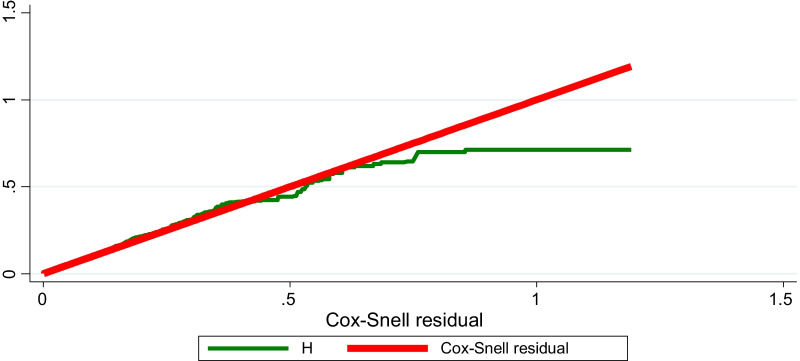
Cox–Snell residuals to evaluate Cox PH model goodness-of-fit, HEW attrition. Ethiopia, 2019

The five variables were well fitted in the final Cox PH model. As shown in Table 3, level four HEWs had a lower hazard ratio of 0.35 [95% CI 0.3, 0.5] than did level three, but the hazard ratio was higher for those with diploma/degree qualifications, at 3.3 [95% CI 2.3, 4.9]. HEWs who had children had a lower hazard ratio, 0.54 [95% CI 0.4, 0.67], than their counterparts. The overall model goodness-of-fit test was determined by the Cox–Snell residual test and was found well fitted, as shown in Fig. 2.

**Table 3:** Crude and adjusted hazard ratios for time to attrition

Variables	Categories	Crude hazard ratio [95% CI]	Adjusted hazard ratio [95% CI]
Certification level	Level 1–3	1.00	1.00
	Level four	0.2 [0.17–0.3]*	0.4 [0.3–0.5]*
	Diploma and degree	2.1 [1.7–2.6]*	3.3 [2.3–4.9]*
Annual leave	No annual leave	1.00	1.00
	1–3 annual leaves	0.6 [0.4–0.7]*	0.84 [0.6–1.1]*
	≥ 4 annual leaves	0.5 [0.4–0.7]*	0.9 [0.7–1.1]
Having biological children	No	1.00	1.00
	Yes	0.4 [0.3–0.5]*	0.5 [0.4–0.7]*
Certificate of competence status	Certified	1.00	1.00
	Not certified	1.9 [1.6–2.4]*	1.6 [1.3–2.0]*
Deployment year	2004–2008	1.00	1.00
	2009–2013	2.0 [1.6–2.5]*	1.5 [1.1–1.9]*
	2014–2019	3.5 [2.4–4.9]*	2.6 [1.7–3.8]*
Family size	1–3	1.00	1.00
	≥ 4	0.5 [0.45–0.65]*	1 [0.75–1.2]
Woreda type	Rural	1.00	1.00
	Urban	1.3 [1.1–1.8]*	0.5 [0.3–0.7]*

* significant association at *P*-value less than 0.05

Ethiopia, 2019

*CI* Confidence interval

### Findings from the qualitative study

Four major themes around reasons for attrition appeared in the analysis of the IDIs and the FGDs: psychosocial factors, administrative problems, incentives and career progression, and work environment.

Psychosocial factors were characterized by HEWs’ lack of skill, personal conflict with officials, personal health problems, lack of community acceptance, family influence, and lack of child care. Administrative factors affecting HEW attrition included a poor performance appraisal system, denial of legitimate annual leave, unresponsiveness of officials, discrimination, disrespect, poor supervision, and not keeping a promise. Inadequate incentives, such as low wages, poor incentive packages, and career problems, including the dearth of educational and training opportunities and lack of recognition, were mentioned as factors that led to the ultimate exit of HEWs. The work environment itself was identified as one of the themes that led HEWs to resign and included reports such as not being able to transfer work locations to the HEWs’ place of birth, the distance of the worksite from the woreda health office, lack of transportation, and difficult topographical conditions for their work.

## Discussion

Attrition during the 15 years of follow-up was 21.1%, the overall incidence of attrition over the 15 years was 3.1%, the median time to exit from HEWs workforce was 5.8 years, and intention to leave among HEWs who were actively on the job during the survey was 39.5%. Being level four certified, having children, and working in urban locations were associated with a lower hazard ratio of attrition than being level three, having no children, and working in rural areas, respectively. Being COC uncertified, diploma/degree holders, or deployed after 2008, however, were associated with a higher hazard ratio of attrition. The qualitative study revealed that four major reasons—psychosocial, administrative, incentive and career problems, and working environment factors—appeared as causes of resignation that explained a majority of the attrition. Furthermore, low salary and career development, psychosocial factors, and workload were the reasons for intention to leave.

HEWs hold a unique position in Ethiopia; they are neither considered to be health professionals nor community health volunteers. HEWs are CHWs paid to be full-time employees, generally recruited from their own localities, with no clear expectation for how long they should serve or where and how they can progress in their future careers. An attrition rate of 3.2% to 77% was observed among community health workers (CHWs) in the 1980s, which may correlate to them being volunteers and lack of incentives [14]. In this study, an attrition rate of 21.1% throughout the course of the program was observed, with an annual attrition rate of 1.5%. This is considered low compared to international recommendations, where a 15% annual attrition rate is a warning sign for program evaluation [32]. Various studies on CHWs have reported a higher attrition rate than in Ethiopia. Reports from Bangladesh, Sri Lanka, Ghana, Kenya, Nepal, and the Solomon Islands reported annual CHW attrition rates of 18.5%, 10%, 8.4%, 7.1% 5%, and 4.8%, respectively [18, 33–37]. This difference may be attributable to incentives and career-development strategies, as well as program follow-up. Similarly, a study in Uganda showed retention rates of 95%, 91%, and 86% during the first, second, and fifth years of implementation, respectively, and concluded that medium-term retention of CHWs is possible [38].

Intention to leave is a risk factor for actual attrition behavior [39]. This study revealed that intention to leave was 39.5%, which was in line with studies done in China and Sweden [10, 11] but low compared with a study conducted in North Wollo, Ethiopia [40]. This difference may be attributable to differences in the sample size and scope of the study areas. As confirmed by this study, various factors contributed to HEW’s attrition and intention to leave. These included personal conflict with officials, personal health problems, lack of community acceptance, family influence, lack of childcare, poor appraisal system and denial of legitimate annual leave. Furthermore, the unresponsiveness of officials, poor support, low payment, dearth of educational and training opportunities, not enough career development opportunities and recognition were also mentioned as contributors. These results were similar to those of studies done elsewhere [13, 39, 41].

The median time to exit from HEWs workforce was 5.8 years. That means that 50% of HEWs leave their posts after serving nearly 6 years. The median time for censored cases was 8 years; there is only a 2-year difference in median service years among censored and attrition cases. This may be correlated with the inadequate career opportunities provided for HEWs, as supported by an Indian study [42]. Another possible reason is related to the recruitment criteria. As the majority of HEWs are unmarried, they may leave their work when they establish a family far from their workplaces. The incidence rate was reported here at 3.1%/PYO, somewhat lower than findings from Kenya [35]. This difference may relate to the type of deployment: Kenya had community health volunteers who did not get paid. The highest incidence, 4.8%/PYO, was seen in the seventh year of implementation. This finding, in addition to median time and the qualitative results, indicated that the program offers weak incentives, such as career development and educational opportunities that lead HEWs to leave after serving an average of 6 years. The literature reveals that inadequate pay, better employment positions in other fields, and the loss of other economic opportunities are the major reasons for attrition among CHWs [14, 35, 38].

Starting in 2014, the government of Ethiopia allowed upgrading of rural HEWs to level four, and the hazard of attrition in this group of HEWs is lower in the current study [AHR = 0.4 (0.3, 0.5)] compared to HEWs who did not have the opportunity to be upgraded and remained as level three workers. The qualitative findings in the current and previous studies [35, 36, 38] show that providing training for CHWs encourages them to stay in their workplaces longer time. Training is also important in developing confidence, attracts others to become HEW, and increases their skill [43, 44]. On the other hand, having a diploma or degree certification was associated with an increased risk of attrition in the current study. In the Ethiopian context, diploma/degree holders are assigned to urban settings, where other job opportunities, with better payment, is common; they are professional graduates with an opportunity to upgrade and run their own businesses in private clinics. The hazard of attrition among HEWs with at least one child was 46% lower than among HEWs with no children. This may be partly explained by the fact that HEWs with children are more settled. In Pakistan, being married and having children were criteria for selecting CHWs, assuming that community trust around this group was relatively high and that their desire to change place is limited [32].

COC certification is mandatory for HEWs to upgrade to the next level. In the current study, those who were not certified for COC had a hazard of attrition 1.7 times higher than those who were COC certified. It is very difficult to expect quality service from employees without future careers or educational opportunities, and it is not surprising to observe an increased hazard of attrition among them. In line with this, the hazard of attrition among HEWs deployed from 2009 to 2013 was 1.5 times higher compared to those deployed in the first 5 years of the program. Similarly, those appointed from 2014 to 2019 had a hazard of attrition 2.6 times higher compared to those deployed in the beginning of the program (2004–2008). Those assigned to urban settings had a lower hazard of attrition compared to those assigned to rural areas, although diploma/degree holders, who are usually assigned to urban settings, had a high attrition rate.

## Conclusion and recommendations

Our findings reveal that the contributing factors of attrition and intention to leave are similar. Although theories suggest that having the intention to leave normally translates to attrition from a position, attrition itself was not as high amongst HEWs, as their intention to leave. This could be because of a shortage of suitable jobs in the public sector for women, who are not highly skilled and have been trained only on specific health interventions that are delivered by HEWs. While attrition rates have largely varied between countries, from an empirical point of view we believe that the rate of attrition of HEWs in the current study was generally quite low, while their intention to leave, compared to other countries, was high. Various factors, collectively described as personal, organizational, and policy-related factors, played a role in their attrition and intention to leave. This implies that there is a need to critically examine policies and guidelines that govern this cadre of health workers and changes need to be made to increase their job satisfaction as well as create adequate opportunities for career development.

## Data Availability

Upon request, data will be made available from the corresponding author.

## Abbreviations

CHW: Community Health Worker
CI: Confidence interval
ODK: Open Data Kit
PHCU: Primary Health Care Unit
SD: Standard deviation
WHO: World Health Organization
COC: Certificate of Competency
HEP: Health Extension Program
HEW: Health Extension Workers
HP: Health post
IQR: Interquantile range
PH: Proportional hazard
PYO: Person-year observation
SNNPR: Southern Nations, Nationalities, and Peoples Region
TVC: Time-varying-covariate
